# Dietary Intake of *Curcuma longa* and *Emblica officinalis* Increases Life Span in *Drosophila melanogaster*

**DOI:** 10.1155/2014/910290

**Published:** 2014-05-22

**Authors:** Shilpa Rawal, Pavneet Singh, Ayush Gupta, Sujata Mohanty

**Affiliations:** Department of Biotechnology, Jaypee Institute of Information Technology, A-10, Sector-62, Noida, Uttar Pradesh 201307, India

## Abstract

Intake of food and nutrition plays a major role in affecting aging process and longevity. However, the precise mechanisms underlying the ageing process are still unclear. To this respect, diet has been considered to be a determinant of ageing process. In order to better illustrate this, we used *Drosophila melanogaster* as a model and fed them orally with different concentrations of two commonly used Indian medicinal plant products, *Curcuma longa* (*rhizome*) and *Emblica officinalis* (*fruit*). The results revealed significant increase in life span of *Drosophila* flies on exposure to both the plant products, more efficiently by *C. Longa* than by *E. officinalis*. In order to understand whether the increase in lifespan was due to high-antioxidant properties of these medicinal plants, we performed enzymatic assays to assess the SOD and catalase activities in case of both treated and control *Drosophila* flies. Interestingly, the results support the free radical theory of aging as both these plant derivatives show high reactive oxygen species (ROS) scavenging activities.

## 1. Introduction 

Life style diseases are wholly or partially attributed to diet and have become major health concern [1–4]. Long term effects of diet may cause various diseases like diabetes and cardiovascular diseases and can also decrease longevity [5–7]. It has also been well documented that mother's or the childhood nutrition can potentially have long term health impacts on an individual [8, 9]. In addition, studies have shown that manipulation of dietary intake by increasing or restricting certain food components affects the aging process and longevity [10–13].

India has been blessed with many medicinal plants and these are in use as traditional medicine in “Ayurveda” since hundreds of years [14, 15]. Many such plant products are included in daily diets especially in the form of spices and other food supplements and are known to be rich in antioxidant, anti-inflammatory, antibacterial, anticancerous, and antidiabetic properties [16]. However, detail about their molecular action and dose specificity is not yet known, attracting researchers to explore more in this field [17, 18].

Understanding the biology of aging and life span extension is one of the prime areas of research and well supported by various theories. Oxygen-free radicals or reactive oxygen species (ROS) which cause aging are one of the principal theories of ageing in recent years [19]. The primary assumption of this theory is that normal antioxidant defense levels are not sufficient, so that some ROS escape elimination causing molecular damage, some of which are irreparable and accumulate with age. If ROS causes aging, then enhanced defense against ROS should reduce oxidative stress, slow down aging process, and ultimately extend the lifespan [20].

To this respect,* Drosophila* has been used extensively in aging and longevity studies due to its short life span and easy growing and handling properties in the laboratory [21–23]. The genome similarity and presence of highly conserved metabolic pathways with eukaryotes including human make* Drosophila* one of the best model organisms for investigating various genetic and metabolic pathways to avail the ground level understanding on various gene-environment interactions and their relation to aging process [24, 25].

The present study aims at understanding the effects of food supplement on longevity using* Drosophila melanogaster* as the model organism and its impact on body weight. During this study, the food supplements used were the fruit (fresh crude extract) of* Emblica officinalis *(common Indian name “amla”) and rhizome (powder) of* Curcuma longa* (common Indian name “haldi,” in English “turmeric”). These two are very familiar plant derivatives in India and are considered to contain medicinal properties. For example, curcumin is a major component of turmeric known to exhibit anti-inflammatory, antitumor, and antioxidant properties [19]. Similarly, gallic acid, gallotannin, ellagic acid, and corilagin are some of the important constituents of amla and are also reported to possess several medicinal properties, for example, cardioprotective, gastroprotective, chemopreventive, anti-inflammatory, antimutagenic and also free radical scavenging, and antioxidant [26, 27].

## 2. Materials and Methods

### 2.1. Drosophila Species Used and Experimental Conditions

A mass culture of* Drosophila melanogaster *was used in this study which was being maintained in the laboratory since 2009 on standard maize-yeast-molasses media consisting of maize flour (17 gms), molasses (16 gms), baker's yeast (5 gms), agar (2 gms), nipagine (2 gms dissolved in 2 mL of ethanol), and water in a total volume of 300 mL and maintained at constant temperature and humidity in the laboratory.

For the control lines, the food media used were without addition of any supplement. While in case of treatments, the regular food media were mixed with either turmeric powder in different concentrations (added per 100 mL of media) or with amla at two different concentrations (20 mL and 30 mL per 100 mL of food media), the concentrations of turmeric in culture media were ascertained from earlier study [21, 28] and from preexperimental optimization conducted in our laboratory. Since the food medium becomes dry at higher concentration of turmeric powder, in order to avoid dryness of the media, the maximum concentration used in this study was 0.7 g per 100 mL of media. Similarly, two concentrations of amla were determined after preliminary optimization to avoid media from being too watery. To maintain the purity, the turmeric powder and the amla juice were prepared on mixture grinding the sun-dried rhizome of* C. longa *and the fresh fruits of* E. officinalis, *respectively.

### 2.2. Setting up of the Experiments

Since maximum emergence of* Drosophila* flies takes place during the morning hours, freshly emerged flies were collected between 9 a.m. and 11 a.m. In order to keep the flies stress free, three male and three female* Drosophila* flies were kept in each food vial in case of both control and treated experiments. The three* Drosophila* couples were transferred to fresh food vials containing respective culture media every five days to avoid the effect of media contamination on the flies and not to mix-up with their offspring. For each experimental condition and control, 36 vials were set up with six flies (three males and three females) in each (total 216* Drosophila* flies) and were kept inside a BOD incubator with constant temperature (24 ± 1°C) and other environmental conditions.

### 2.3. Observations

#### 2.3.1. Life-Span Days

All vials were kept under constant observation and the life span of each fly was noted by simply noting the survivability of flies which includes the number of days from the recorded birth to death. The mean life span (*X*_*m*_ = ∑*x*_*i*_/*n*, where *X*_*m*_ is the mean lifespan, *x*_*i*_ is the lifespan of *i*th fly, and *n* is the total number of flies in the sample) was calculated in each case. Maximum and minimum days of life span in case of each experimental condition were noted down to study the range variation.

#### 2.3.2. Body Weight

The weight of single fly was recorded at 10, 20, and 30 days interval during their lifespan in both sexes separately in case of both control and treated flies.

### 2.4. Enzymatic Assays

#### 2.4.1. Preparation of Crude Extract

The* Drosophila* fly crude protein was prepared at 4°C to prevent the enzymes from being degraded. Six flies were homogenized in the lysis buffer which contained 50 mM phosphate buffer and 0.1 mM EDTA in a total volume of 800 *μ*L. The lysate was then centrifuged at 10,000 g for 10 minutes and the supernatant forms the crude extract [29].

### 2.5. Superoxide Dismutase Activity Test by NBT Assay Method

The test mixture, consisting of 50 mM potassium phosphate (pH 7.0), 0.1 mM EDTA, 75 *μ*M NBT, 2 *μ*M riboflavin, and 50 *μ*L protein sample in a total volume of 2.5 mL, was placed below a 15 W light source and the reaction was allowed to run for 30 min, during which time the color developed. The reaction was stopped by switching-off the light and the tubes were covered with a black paper [30–32]. The absorbance was observed at different time points at 560 nm in a spectrophotometer to determine the enzymatic activity. The rate of increase in absorbance units (*A*) per minute for the negative control and for the test samples is determined by the formula given as (1) and the percentage inhibition for each sample is calculated using the formula given in (2):

(1) $$\begin{array}{c} \frac{A_{560\;\text{nm}}\text{final}-A_{560\;\text{nm}}\text{initial}}{\text{Time}\;\text{Interval}}=\frac{\Delta A_{560\;\text{nm}}}{\text{minute}} \end{array}$$

(2) [(ΔA560 nm/minute(−)control)−(ΔA560 nm/minutetest)]ΔA560 nm/minute(−)control  ×100=%  Inhibition.

### 2.6. Superoxide Dismutase (SOD) Activity Test by Native PAGE

Native PAGE was carried out on 12% nondenaturing gel. SOD was located on native PAGE by soaking the gels in 2.5 mM (NBT) for 20 minutes followed by a 15 minutes immersion in 36 mM potassium phosphate buffer (pH 7.8), 28 mM TEMED, and 28 mM riboflavin. Bands of SOD were observed after exposing the gels to light for 5–10 min, till the bands appeared on a dark background [32].

### 2.7. Catalase Activity Test (CAT) by H_2_O_2_ Assay

Hydrogen peroxide solution (59 mM H_2_O_2_ dissolved in 50 mM potassium phosphate buffer, pH 7.0) was added to protein samples (50 *μ*L each time) and the CAT activity was determined by the oxidation of H_2_O_2_ at 240 nm where one unit of activity is defined as 1 *μ*L of H_2_O_2_ decomposed per minute, considering the* molar* extinction coefficient of* hydrogen peroxide* (62.4). Readings were taken at different time points to determine the activity of the enzyme [33].

### 2.8. Statistical Analysis

The mean life span and the standard errors (SE) were calculated for each experimental condition using the XLSTAT computer program. To determine differences in measured variables among experimental groups, a one-way analysis of variance (ANOVA) was employed and statistical significance of the ANOVA tests was determined.

## 3. Results and Discussion

Nutrition plays a critical role in overall health of an organism. However, both under- and overnutrition may seriously impact long term health and life expectancy. Therefore, the study on dietary nutraceuticals has become challenging and fascinating demanding greater attention than before [3].* D. melanogaster *is an excellent model organism to study aging due to its short generation time and lifespan [22]. In the present study, we have exposed* D. melanogaster *flies to food media supplemented with various concentrations of turmeric and amla to evaluate their effect on lifespan and body weight. Furthermore, any adverse effect on the physiology of the organism was also determined.

**Figure 1:**
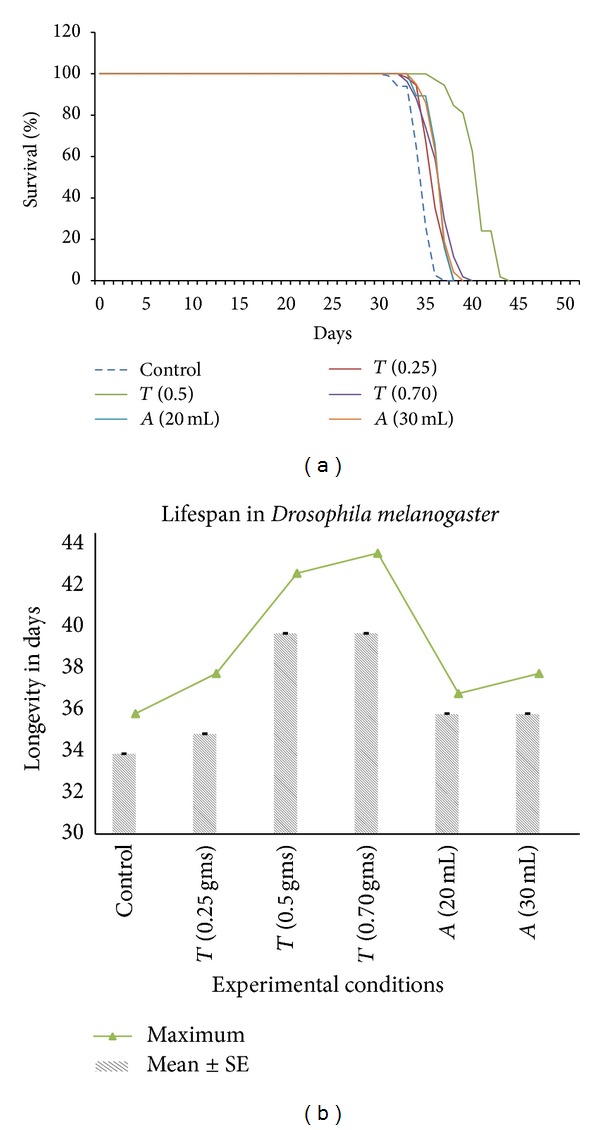
(a) Survival curve for both control and exposed* Drosophila* flies. (b) Mean ± SE and maximum life span days in control and experimental* Drosophila* flies.

When* Drosophila* fly food was supplemented with turmeric (0.25 g, 0.5 g, and 0.7 g per 100 mL of media), there was a marginal increase in lifespan at a lower concentration (0.25 g/100 mL). Interestingly, with increased concentration of turmeric in food medium, the life span also increased (Figures 1(a) and 1(b)) until a point of concentration, beyond which, increase in the lifespan was not that prominent. The observed effect might be due to the absorption threshold of turmeric, and higher concentrations may not get absorbed completely, thereby, having no further effects on the lifespan. Similarly, intake of amla (at two different concentrations) also increased the life span of* D. melanogaster*, but less effectively as compared to turmeric (Figures 1(a) and 1(b)). The average and the maximum life span for each control and exposed* Drosophila* flies were analysed and the survival curve shows day-wise percent of survival for each experimental conditions as well as control (Figures 1(a) and 1(b)). When the turmeric concentration of 0.5 g/100 mL was added as food supplement, the life span of flies could be increased to as high as 42–44 days in comparison to any other experimental conditions, indicating the action of these medicinal plant products in a dose-dependent fashion. The results of all the experimental and control conditions were compared with each other using one-way ANOVA and variation was found to be statistically significant (at *P* < 0.001) in each case (Table 1).

**Figure 2:**
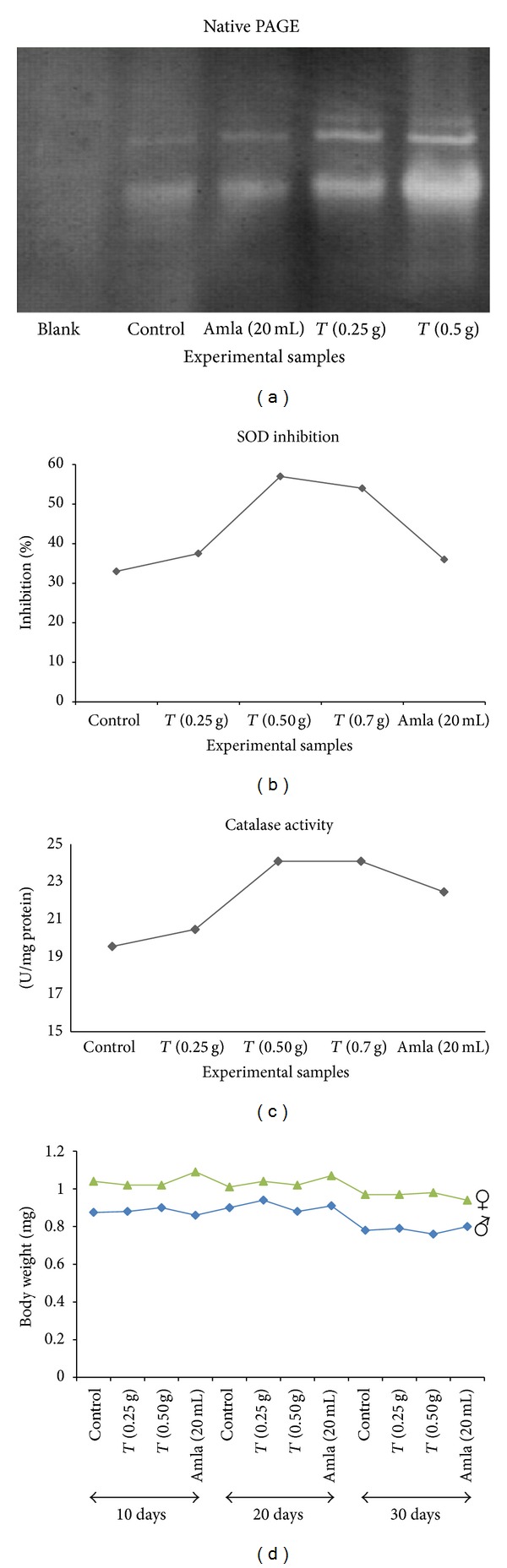
(a) Native PAGE of control and experimental samples showing differential expression of SOD. (b) % inhibition of the rate of increase of absorbance at 560 nm due to reduction of NBT by superoxide radical. A strong inhibition corresponds to increased activity of SOD. (c) Graph showing catalase activity as U/mg of different samples. (d) Body weight of single* Drosophila* fly at regular interval of 10 days in control and treated conditions.

The present findings on the effect of these two medicinal plant products corroborate with the results of previous study to some extent. Although there is only one recent report available in literature showing positive effect of amla (in form of drug) on life span and reproductive fitness of* Drosophila *to certain extent [34], it has also been earlier reported that dietary supplements of curcumin (a component of turmeric) extends lifespan of* D. melanogaster *[21]. Since the mechanism by which curcumin enhances life-span in* Drosophila* is unknown, in order to understand such mechanism, we performed the enzymatic assay of superoxide dismutase (SOD) and catalase enzymes. It is a well-known fact that the antioxidant enzymes, for example, SOD, catalase, and glutathione dependent enzymes, get induced as natural defense mechanisms against oxidative stress in biological systems [35]. For example, SOD, a group of metalloenzymes, facilitates the removal of hydrogen peroxide (H_2_O_2_) on to molecular oxygen and water [35]. Overexpression of enzymatically active bovine SOD in* Drosophila* flies has been shown to confer resistance to paraquat, an (O2−)-generating compound, and also increase the mean lifespan of several of the transgenic lines [36]. In another experiment, expression of Cu/ZnSOD overexpression extended the mean life span of flies up to 48%, while catalase had no significant effect [37], although it increased resistance towards exogenous hydrogen peroxide, paraquat and cold stress [35]. However, overexpression of catalase in mitochondria, nucleus, or peroxisomes has been shown to increase lifespan of mice [38, 39]. CAT enzyme plays crucial roles as antioxidants and constitutes the primary defense system against the toxic effects of superoxide radicals (O2−) in organisms [40]. According to the most acceptable free radical theory of aging, the potentially toxic-free radicals, primarily the ROS, superoxide, and hydroxyl radicals generated during aerobic metabolism, are inactivated or scavenged by antioxidants before they can cause damage to lipids, proteins, or nucleic acids [40]. To test this hypothesis, the* Drosophila* fly protein samples of each experimental condition were tested for SOD and CAT activities. The results indicate that turmeric significantly increases the activity of total SOD at optimal concentrations, as determined by the nitro blue tetrazolium (NBT) reduction assay and native-PAGE (Figures 2(a) and 2(b)). Marginal increase on the activity of SOD was also observed in case of flies fed with amla supplemented food in 20 mL/100 mL concentration. Since increase in the life span seems to be correlated with the increase in the SOD activity, it can be argued that scavenging of the superoxide radicals might be a major factor in increasing the lifespan of* Drosophila *flies in the test conditions. Increased activity of CAT enzyme was also observed in flies supplemented with both turmeric and amla (Figure 2(c)), indicating antioxidants properties of these two medicinal plants. These two medicinal plants do not seem to have any adverse effect on the flies, as no significant change in the body weight with age of the medicinal plant-supplemented* Drosophila* flies could be found. Declining of the body weight after 30 days (also observed for the control set of flies, Figure 2(d)) justifies the above contention. However, amla and turmeric might not have been the sole factors in increasing the life span of the* Drosophila* flies as there might be other (unknown) enzymes and target factors involved on which turmeric and amla have an effect.

In conclusion, the results presented in this work support the hypothesis that intake of* C. longa *(curcumin) and* E. officinalis *(amla) increases life span in* D. melanogaster.* Very similar observations have been made in another experimental model organism,* Caenorhabditis elegans* [41]. The life span is significantly increased in* D. melanogaster *flies exposed to both turmeric and amla supplemented food, which is presumed to be due to their high antioxidant properties [28] as evidenced from both SOD and catalase enzymatic assay. However, the present observations reveal antioxidant properties and the ROS scavenging activities of amla to be lower than turmeric. In both cases, oxidative stress reducing ability was found to be dosage-specific. Additionally, it has been observed experimentally that the intake of both these antioxidants has no significant effect on body weight in* Drosophila* flies. The slight decline in the body weight in the later life (at 30 days) may be due to the old age of the flies. The present study therefore could demonstrate that turmeric and amla increase lifespan of* Drosophila*. However, further research is needed to underpin the exact component(s) with a role in increase of life span in* Drosophila*. Though few studies have been conducted with exposure to both plant products in certain disease conditioned mammals (diabetic, cancer, etc.) including human, no such reports are available on aging process and on its underlying mechanism [26, 42]. Considering that* Drosophila* has served as a “model organism” for biomedical research, such knowledge could be well implemented for detailed understanding on the role of diet in ageing process in humans.

## Figures and Tables

**Table 1:** Summary table of one-way ANOVA for differences in life span days among control and experimental flies.

ANOVA																				
Source of variation	Control versus *T* (0.25 gm)				Control versus *T* (0.5 gm)				Control versus *T* (0.7 g)				Control versus amla (20 mL)				Control versus amla (30 mL)			
	SS	df	MS	*F*	SS	df	MS	*F*	SS	df	MS	*F*	SS	df	MS	*F*	SS	df	MS	*F*
Between groups	190.67	1	190.67	142.14*	3745.33	1	3745.33	1642.26*	3657.52	1	3657.52	1830.23*	350.28	1	350.28	290.66*	377.81	1	377.81	306.47*
Within groups	576.81	430	1.34		980.66	430	2.3		859.31	430	1.99		518.19	430	1.20		530.10	430	1.23	

Total	767.48	431			4726	431			4516.83	431			868.47	431			907.92	431		

*The *P* value for this statistics is *P* < 0.001.
